# Experiences and Challenges of Health Professionals in Implementing Family-Centred Planning: A Qualitative Study

**DOI:** 10.3390/children11010132

**Published:** 2024-01-22

**Authors:** Lorena Cuenca-Sánchez, David Sánchez-Teruel, Maria Auxiliadora Robles-Bello

**Affiliations:** 1 Department of Psychology, University of Jaen, 23071 Jaen, Spain; lcs00002@red.ujaen.es; 2 Faculty of Psychology, University of Granada, 18012 Granada, Spain; dsteruel@ugr.es

**Keywords:** early childhood care, family-centred planning (FCP), professional perceptions, health services, health personnel

## Abstract

Early childhood intervention is crucial for the development of minors with disabilities or at risk. Family-centred planning (FCP), which involves families in care, stands out in this context. Despite its importance, little is known about professionals’ experiences of its implementation. FCP aims to tailor services to the needs of the family and the child within the Spanish health system. This study highlights the importance of assessing professionals’ perceptions of FCP. Professionals rooted in traditional approaches may resist change. To assess the implementation of FCPs, the study explores the perspectives of 25 healthcare professionals using qualitative methods to assess their experiences. The qualitative descriptive phenomenological design, following Giorgi’s modified Husserlian approach, seeks to understand the essence of the phenomenon from the participants’ perspective. Two main themes emerged: (1) a social and work organization that perpetuates rehabilitation or early stimulation practices and (2) a socio-family and work organization that promotes FCP adherence, along with subthemes and units of meaning. The evaluation reveals common challenges, such as the need for solid training and institutional support. Evaluating the experience of professionals is essential to overcome barriers and ensure the successful implementation of FCPs. Administrators have an important role to play in providing social, health, and educational alternatives.

## 1. Introduction

Early childhood care is an essential area in the development of children with, or at risk of, developmental disabilities. In this context, family-centred planning (FCP) has emerged as a fundamental approach that aims to involve families in the care process and provide comprehensive support for children [1]. However, despite the growing importance of FCP in early childhood services, knowledge about the experiences of professionals working in these services in relation to the implementation of FCP remains notoriously limited [2].

Family-centred planning (FCP) is an important approach to early childhood care that emphasizes collaboration between professionals and families [3], recognizing the uniqueness of each child and their circumstances. Over the years, several studies have underlined the importance of this approach in improving the quality of life of children and their families, highlighting its ability to strengthen collaboration and partnership [4]. This strategy is based on the fundamental principle that families are experts in their children’s lives and should be active partners in making decisions that affect their development. The FCP recognises that each child is unique and that their needs can vary significantly, underlining the importance of personalizing early childhood services. Despite its potential to improve the quality of care, the implementation of FCP in early childhood settings faces a number of challenges, including institutional and professional barriers [5].

Family-centred planning is an innovative approach that has gained recognition in recent years as a valuable framework for the early intervention of children with, or at risk of, disabilities and developmental disorders. FCP has its origins in the philosophy of the ‘person-centred approach’, which was developed in the 1980s as an approach to the provision of support services for people with developmental disabilities [6]. The essence of FCP is the belief that the quality of care improves when families play an active role in decision-making and service planning. Early intervention and health professionals work in partnership with families, recognizing the experience and knowledge that families have about their children [7].

A fundamental component of FCP is the individualized planning process, which involves the identification of goals and objectives for the development of treatment sessions that professionals design for children, as well as the selection of appropriate interventions and services. This process is based on the creation of a map of content to be worked on and discussed with the family, reflecting the family’s aspirations and expectations for their child’s future [3]. FCP also fosters a relationship of partnership and respect between professionals and families, leading to a more effective and empathetic supportive environment. Benefits of FCP include improved access to services and resources, family satisfaction, and increased parental involvement in the decision-making process [8,9].

Despite these benefits, the implementation of FCP in early childhood centres is not without its challenges. The transition from traditional models of care to more family-centred approaches requires changes in organizational culture and working practices. In addition, health and education professionals may experience resistance or difficulty in adopting new approaches [10]. The implementation of FCP is hampered by institutional barriers to the adoption of family-centred approaches in early childhood centres. In many cases, organizational structures and policies are designed for a more traditional, hierarchical model of care. Bureaucratic processes, rigid rules and regulations, and a lack of flexibility can prevent professionals from having the autonomy to tailor services to the individual needs of each child and family. In addition, the lack of financial and human resources can limit the capacity of early childhood centres to provide personalized services [11].

Professional barriers are also a major challenge to the implementation of FCP. Early childhood professionals may be used with more traditional approaches based on medical or special education models. Moving to a family-centred perspective may require a reevaluation of professional practices and adaptation to new ways of working. Some professionals may feel resistance to change, fear of loss of control, or anxiety about a lack of knowledge to effectively meet the needs of children and families [12]. Training and professional development are essential to overcome these barriers.

Cultural barriers can also hinder the implementation of FCP. Cultural differences between professionals and families can lead to misunderstandings and conflicts in decision-making and service planning. Lack of cultural sensitivity and cultural competence can be significant barriers to building effective and trusting relationships with families [13]. FCP requires a culturally sensitive approach to ensure that services are culturally appropriate.

Given the importance of successful implementation of FCP in early childhood settings, it is essential to assess the perceptions of professionals working in this field [14]. Understanding what professionals think and feel about FCP can shed light on the barriers and facilitators that influence the adoption of this approach. Qualitative research can play a key role in this process, allowing researchers to explore the views and experiences of practitioners [1].

One of the most interesting and challenging aspects of evaluating FCP is to consider the perspectives of the professionals involved in its implementation. These professionals play a crucial role in the process, as they are responsible for facilitating and supporting family-centred planning [3]. Their knowledge, attitudes, and practices influence the experiences of families and people with disabilities. Therefore, understanding what professionals think and feel about FCP is essential to improving its implementation.

Assessing the perspective of practitioners can shed light on critical issues [4], such as the barriers they face in adopting this approach, perceived benefits, difficulties in implementation, and training needs. In addition, it can provide valuable information for the design of training and support strategies to strengthen the successful implementation of FCP.

However, the existing literature still provides only a partial picture of professionals’ perspectives on FCP. The evaluation of their views and experiences is still a developing field with great potential to contribute to the continuous improvement of care and support for people with disabilities and their families. There is a lack of studies that explore in depth the experiences of professionals who put FCP into practice. This is particularly important as these professionals play a crucial role in the effective implementation of FCP and, thus, in improving the quality of life of children in early childhood centres.

As can be seen, little is known about the experiences linked to family-centred planning of professionals working in early childhood centres. The aim of this study is to describe and report on the experiences of early childhood professionals working with families of children with developmental disorders or at risk of having developmental disorders who are cared for in early childhood centres on how they implement family-centred planning.

## 2. Method

### 2.1. Design

This study used a qualitative descriptive phenomenological design conducted using the modified Husserlian approach of Giorgi’s [15]. The phenomenology approach seeks to describe the essence of a phenomenon as lived by a person who had the experience and to comprehend the meaning of this experience from participants’ perspectives [16]. This design was chosen because the aim is to describe and report on the experiences of professionals in early childhood centres working with families of children with developmental disorders or at risk of having such disorders who are cared for in these centres.

### 2.2. Participants and Context

The study was carried out in 5 early childhood centres in southern Spain. Convenience sampling was used to recruit participants. The inclusion criteria were as follows: (1) working in an early childhood centre for at least 1 year; (2) not having incurred any administrative proceedings for any reason; (3) not having been dismissed from the service due to mental health problems; and (4) being a professional in clinical speech therapy, psychology, or physiotherapy with training in ECI. Thirty-five people were initially contacted, of whom 27 were eventually interviewed and volunteered to participate. Two of the first interviews were used as pilot interviews that were not used for the analyses of this study. Finally, there were 25 participants.

### 2.3. Data Collection

The data were collected from May to September 2023, following ethics approval. The participants were interviewed face-to-face using an in-depth interview method through a semistructured interview (Table 1). Following the phenomenological methodology, open questions were used to provide ample freedom of response for the participants, and secondary questions were used with the aim of understanding more about the professionals’ experiences. Interviews were conducted by MARB, a psychology teacher with training in qualitative methodology who had previous training in qualitative interviewing. A pilot test with two participants was conducted to adjust the interview guide, but they were not included in this study.

Interviews were individual and a couple. Thus, to protect the privacy of the participants and ensure a comfortable and quiet environment for the interview, the author conducted it in a room in the department. The interviews varied in length from 40–65 min, with an average duration of 50 min. The interviews were audio-recorded and transcribed verbatim, and they were kept strictly confidential.

Data and results obtained in this study were regularly evaluated in meetings with the professors and researchers of team research, who were experts in qualitative studies with thorough continuous reflection. The transcripts were reviewed by the team researchers to verify their accuracy. It was not necessary to repeat interviews with the same participants in this study. It was assumed that saturation was reached when the researchers were sure that no more data codes related to the questions and no more information was forthcoming. Regular meetings with the team researchers were done to discuss data saturation, transcription, and repeat themes. Data collection was stopped as soon as it was observed that no new information emerged in the analysis process, thus reaching the principle of data saturation [17].

### 2.4. Data Analysis

Phenomenological methods were used to analyse the data [15,16]. First, the transcripts were reread several times to capture their overall meaning. Then, each document was divided into units of meaning in relation to the aim of the study. Next, the units of meaning were grouped into subcategories related to their content. Next, richer and more complex descriptions were sought based on more semistructured interview questions, such as “What do you mean by this experience?” In addition, the interviewer’s questions and comments were limited to requests for clarification or elaboration and reflections on what the interviewee had already said. Many of the subcategories’ units of meaning were revised and consequently rewritten, eventually giving way to a more coherent description. Finally, differences and similarities between the interviews in the different transcribed documents were sought to further refine the structure of the analysis. ATLAS.ti software (version 8.0) was used to facilitate the analysis. Finally, the research report was drafted, and the most illustrative quotations and the most eloquent examples were selected in relation to the stated objective (Table 2).

### 2.5. Rigour

The participating researchers made judgements about the robustness of the research conducted in terms of the appropriateness and transparency of the methods used. Methodological rigour was ensured at each stage of this research in order to achieve trustworthiness and to ensure the credibility, transferability, dependability, and confirmability of the results [18].

### 2.6. Ethical Considerations

This study was conducted in accordance with the ethical guidelines of the Declaration of Helsinki. It was approved by the Ethics Committee of a Spanish public university (code: JUN.23/3 PRY). Participants were informed of the aim of the study and of the voluntary nature of their participation. They were informed that their answers would not affect their work, given the anonymous nature of the data processing. Signed informed consent was obtained from all participants before data collection began, explaining the purpose and nature of the study and assuring them that they could withdraw from the study at any time without negative consequences.

## 3. Results

The main characteristics of the 25 people who participated in this study are summarized in Table 1. Participants had a mean age of n = 31.84 (SD: 41.36%), with the majority being female. The mean number of years working in this type of centre was 5.08 years (SD = 8.79) (Table 3).

Two main themes were extracted, namely (1) a social and work organization that maintains early rehabilitation or stimulation practices and (2) a socio-family and work organization that promotes FCP follow-up, which together with their subthemes and units of meaning (Table 4) can help us to describe and inform the experiences of early childhood professionals working with families with children with or at risk of developmental disabilities who are cared for in these centres in terms of how they implement family-centred planning.

### 3.1. An Organizational Structure and Work Environment That Maintains Practices of Rehabilitation or Early Stimulation

This topic details three aspects on which professionals or technicians from different fields of work (physiotherapy, psychology, and speech therapy) reported their experiences and the construction of knowledge of the reality that began with the conscious awareness of the different agents involved in an early childhood intervention program. On the one hand, the users receiving treatment—in this case, the parents of children with developmental disorders or at-risk children—who are served in an early intervention centre are studied. On the other hand, the professionals themselves and the socio-health and educational context related to administration are discussed here.

#### 3.1.1. Conditions of the Person Receiving Early Childhood Intervention Services That Lead to Maintaining a Rehabilitative Model

This study refers to what professionals think about the personal situation of parents who are users of early childhood intervention centres, which leads them to focus on the rehabilitative treatment of children with developmental disorders or at risk as a better option for their children’s treatment in contrast to FCP. Participants describe how parents attend sessions distressed to start treatment as soon as possible without knowing exactly what to do or why they are there, how they become desperate when they think that explanations make them lose time, and how they should be working on activities with the child with a developmental disorder or at risk. The worse the physical and mental health deterioration of the child, the greater the distress of the parents and the desire (with a significant amount of anxiety) for someone to do something for their child.

*“…parents come to the centre with high expectations. The paediatrician refers them to the centre because, at the medical level, nothing can be done for the child since the development is compromised and needs stimulation in different areas. When they arrive at the centre and realize that there are no doctors, that the treatment is long-term, and that they are being informed about disability, it becomes a significant burden that they must digest.”*

(p. 14).

Participants also described, among other aspects, the importance of the initial contacts or sessions with families. When delivering news about a specific developmental disorder diagnosis, it was experienced as true mourning for the loss of the idea of a perfect child. In these situations, two different realities are described: the child on the one hand and the family on the other. Participants understand that both realities need to be addressed. The child, based on their diagnosis, will have a specific treatment along with a timely evaluation. Simultaneously, the family requires attention, even more so, or in parallel, because the news itself leaves them traumatized. Participants express that they felt a responsibility to assist these families because they had to react for two reasons: for themselves and because their reaction would decisively influence the future of their children. However, the latter was still unknown, and they were concerned about aspects that were not relevant, as in the following case.”

*“…I receive many complaints about the schedules assigned to the children’s sessions. I constantly undergo adjustments and changes to try to help with family reconciliation. Some families are very flexible in this regard, while others are uncompromising.”*

(p. 15).

Professionals narrate that they fear situations in which they have families and that, when the individualized work program for the child does not yield the expected results, it is common for the responsibility to be placed on the professional or to blame the professional for the lack of progress in the child. They caution in their narratives that one must be careful with the behaviours of overprotection by parents, non-acceptance, and the delegation of responsibilities to technicians. They hear typical phrases that already raise suspicions that parents have not fully accepted the situation they have to live through and that they are unhealthy for the work objective.

*“…he told me that since there was no one else as prepared as I was to make his children read, that he had spoken to other parents, and that I was the best… What burden he placed on my shoulders. I didn’t know how to respond. What could have initially been a compliment made me feel very uneasy.”*

(p. 17).

#### 3.1.2. Working Conditions That Lead to Maintaining the Rehabilitative Model

It refers to the personal and professional situation of technicians from different areas of work in early childhood intervention centres that leads them to believe that it is better to focus on the treatment of the child with a developmental disorder or at risk as the most suitable option based on the cost–benefit evaluation of the work situation.

*“…this job is not well-paid. I have a lot of responsibility, and I even have to attend training on weekends”*

(p. 1).

When participants in our study talked about their work, they felt a significant burden of responsibility because it involved a crucial commitment to shaping the life of a child. As a result, stress was sometimes generated, hindering clear thinking and action when facing the demands of parents to share decisions about the treatment. The same happened when they had to attend to children with complex issues they were not familiar with. On all those occasions, they preferred that parents not enter the sessions since the interaction with the child was always more satisfying than with the parents.

*“…I don’t have time to talk to parents. I tell them that if they want to talk to me, they must request it in advance and on a day when they don’t bring the child because it’s not possible to talk calmly with the child around. Therefore, parents settle for what I provide in writing in the work report. With that, they already know what I’m working on, and if not, they can stand behind the mirror instead of going for coffee.”*

(p. 8).

There were also professionals who presented a narrative lacking self-criticism, feeling like owners of the truth. They were convinced that the families they visited were fortunate, unlike other families. Proud of their training and years of experience, they were capable of promising to bring the child forward if their guidelines were followed literally, based on a working methodology of ‘everything for the family, but without the family’. Consequently, when something went wrong, blame was placed on the parents. Additionally, they expressed that they liked working in a team under the umbrella of an interdisciplinary methodology, but at times, it became a source of conflict among colleagues because they could not reach agreement on common work objectives.

*“…the child is assessed, their objectives are programmed, new schedules are established. The good thing about all this is that with the arrival of more children, I now have more hours in my contract. The latest child who has entered is going to stay for a long time, and the burden will be taken on by the psychologist.”*

(p. 15).

#### 3.1.3. Shortcomings in the Socio-Healthcare and Educational Systems

The participants highlighted a series of conditions and circumstances within the social, cultural, and institutional framework that lead families receiving care in early intervention centres to perceive the rehabilitative treatment model for their children as the sole option, unaware of alternatives. This may be attributed to factors such as the fact that early childhood care is still not standardized across the Spanish territory; some autonomous communities address it through social services, while others, like the autonomous community of Andalusia, approach it from the health sector.

In many regions, early childhood care is managed through grants provided by the administration, contingent on treatment hours without a review of healthcare quality. There are still laws to be implemented, a lack of economic resources, a shortage of specialized professionals, waiting lists for children who, by definition, should be attended to as soon as possible, impossible schedules, a gap between legislative and scientific recommendations, significant regional differences regarding the rights of families and professionals, and sometimes directors of these centres who take on responsibilities in managing facilities and associations. These directors may be frustrated and recognized for their tendency to blame all professionals and technicians they encounter, championing formidable social revenge. Certain whims and mistakes by some directors could be explained and forgiven due to their roles as parents rather than managers.

*“The ‘umbrella of disability’ encompasses a paternalistic, protective, and pitying attitude towards individuals with the capacity to make decisions in this realm—far removed from a truly effective approach based on rights rather than charity…”*

(p. 4).

The participants asserted that coordination between early intervention and various socio-health and educational contexts addressing the same child is crucial for implementing a common working program, ensuring that all stakeholders treating the same individual from different perspectives are aligned. Additionally, they acknowledged encountering healthcare or education professionals who, in their meetings, revealed a lack of conviction regarding the vital importance of the early years of life and the need to intervene as soon as it is discovered that something is amiss in the child’s development. They emphasized that a swift response is crucial during the early years for overall future well-being in personal, family, and social aspects. This reluctance to recognize the vital significance of early intervention can lead to highly dangerous consequences, such as feelings of guilt and failure, irreversibility of neurological consequences, and delays in the acquisition of behaviour, all of which hinder the proper development of the child.

*“One must continue to advocate for medical professionals who fail to conduct the necessary medical tests, indicating that if something needs to be addressed, it will be done when the individual is more mature and that our actions are unnecessarily alarming parents. Fortunately, occurrences of this nature are becoming less frequent.”*

(p. 7).

### 3.2. Socio-Family and Work Organizations That Promote the Implementation of Family-Centred Planning

According to the conducted interviews, participants explain how both families and their own demands, along with those of the administrative system, can support or promote the implementation of a series of strategies to improve the relationship and participation in early childhood intervention programs linked to family-centred planning. On the one hand, participants defined strategies used by family members aimed at the family’s well-being and/or the emotional support of attending parents. On the other hand, professionals define their own responsibility in advocating for and following family-centred planning. Lastly, they also discuss the significant role of the administration in setting a work philosophy to be followed in early childhood intervention. From a preventive perspective, the Public Health System is legislated with the aim of promoting optimal development and maximum personal autonomy for the minors served in these programs, who may have developmental disorders or are at risk of developing them. The goal is to minimize and, if possible, eliminate the effects of any impairment or disability, as well as the onset of additional disabilities, while facilitating the family’s integration into society and enhancing the quality of life for the child and their family.

#### 3.2.1. The Role of the Family in Advocating for and Preserving Family-Centred Planning

Participants define the family’s role as a set of strategies that can be employed by parents or within the broader family context (such as grandparents, etc.). These strategies are utilized either to request family-centred planning or to continue maintaining it. Importantly, these strategies focus on enhancing relationship and participation strategies in early childhood intervention programs. In many cases, families may not be aware that what they are doing aligns with family-centred planning.

The participants shared that many parents actively participated in the treatment program. They perceived themselves as valuable contributors when planning the work report for the upcoming quarter, acknowledging that their assistance is invaluable. Parents provide substantial information about the progress of the work items, and only they can communicate if something is not going well or if the objectives need to be changed in a timely manner. Many decisions need to be made, and having parents on board is always very positive. Additionally, numerous parents sought help in managing the new situation they found themselves in due to their children’s disorder. They needed to share their feelings and requested support to learn how to navigate this situation.

*“…I remember the words of a mother who, from the very beginning, came to the conclusion that she could only help her daughter if she herself was well. She said, ‘This is not a cold that can be cured with a pill or three days in bed. This requires teamwork, and we all need to come together to improve the development of child…”*

(p. 9).

**Figure 1:**
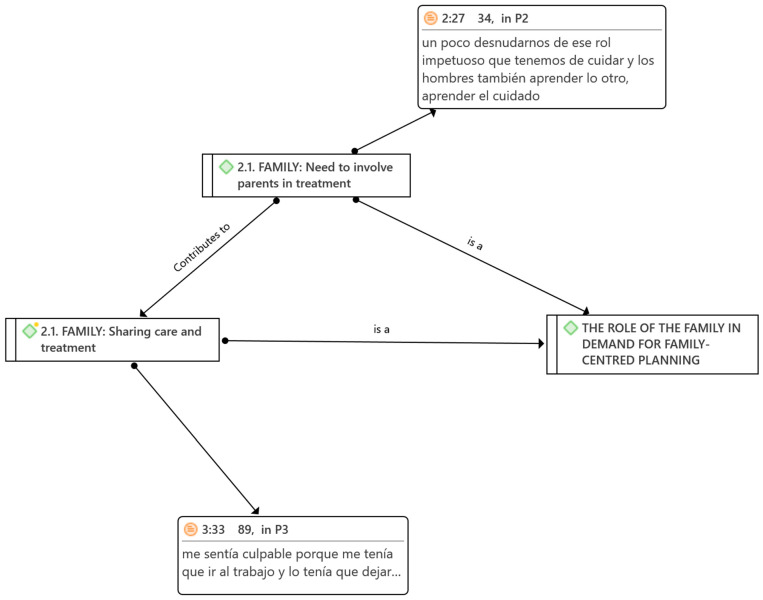
Promoting a cultural shift in childcare, with both parents sharing in the treatment, can benefit the FCP model. Note: It translates the content of the participants (P). P2 mentions that women need to shed the impulsive role of caregivers, and men should learn the caregiver role. P3 indicates feeling guilty because they had to go to work and leave their child.

The participants also described, among other aspects, that there seemed to be a cultural shift underway, albeit in its early stages, as the burden of care and treatment is predominantly borne by mothers (see Figure 1). They are the ones who mostly attend sessions, facing significant challenges in family reconciliation. In many cases, mothers are the ones who stop working to take care of their children who are attending early childhood intervention centres. When fathers do attend, efforts are made to gather more information from them. However, participants expressed concerns that fathers often do not contribute insights into the work or activities conducted at home, as mothers typically handle those responsibilities. There are a few cases in which fathers share the care and treatment of their children. Fathers justify this by stating that, in the division of tasks, their responsibility lies in providing financial support and working throughout the day to ensure their family lacks nothing.

#### 3.2.2. The Role of the Professional in Advocating for and Preserving Family-Centred Planning

The participants referred to the strategies and attitudes they identified in their narratives as key elements that supported or fostered FCP. Participants described their work roles, explicitly highlighting aspects related to family care and the broader context, in addition to the child with a developmental disorder or at risk of having one. Early intervention professionals were aware that they worked closely with the child’s family to understand their needs, goals, and preferences. The professional became a key collaborator for the family, providing information, support, and necessary resources for the child’s development and well-being.

The role of the early intervention professional in improving family-centred planning involved several responsibilities and actions that participants were highly aware of and revealed. This included early assessment and detection and conducting a comprehensive assessment of the child’s development using appropriate tools and techniques. This helps identify any delays or special needs that require attention. The professionals offer counselling and guidance to the family on supporting the child’s development at home and in natural environments, providing specific strategies and activities to promote progress in areas such as language, motor skills, cognition, and social skills. Collaboration with other professionals and services was emphasized. Individualized plans were developed based on assessment and collaboration with the family. Lastly, the professional plays a significant role in coordinating the services and resources necessary for the child and the family.

*“When I don’t know where to turn or what type of activities to offer a child, it’s best to have a team to share all your frustrations with and provide material or ideas to continue intervening in the most effective way.”*

(p. 15).

Paradoxically, to carry out all those functions that participants were aware of, a significant work effort was required to cover everything. This was often expressed more as a wish than a reality. The results of these interviews indicate that actual practices are less family-centred than desirable. At the same time, the desired practices are not fully aligned with FCP, although participants recognized them as desirable practices.

*“I have worked in early childhood intervention centres for a long time and have witnessed the shift in the theoretical paradigm that guides our work philosophy. Currently, I would like the aspects that need to be addressed for the optimal development of children to become a reality. However, I also wish that all this work did not depend solely on me as a professional.”*

(p. 13).

#### 3.2.3. The Role of the Administration: Socio-Sanitary Alternatives to Maximize Family-Centred Planning

The public administration plays a crucial role in maximizing FCP in early intervention programs by providing socio-health and educational alternatives through timely legislation. First, through socio-health services, the administration ensures that children and their families have access to comprehensive care that encompasses both medical aspects and social resources. This involves facilitating coordination among different healthcare professionals, such as doctors, therapists, and child development specialists, to ensure an effective multidisciplinary intervention. Additionally, access to social support services, such as psychological counselling and family guidance, is promoted, contributing to the emotional and social well-being of the family.

*“By collaborating with teachers and physicians, we have been able to share relevant information, establish common goals, and adapt our interventions efficiently. This coordination has allowed us to address the child’s needs in all aspects of their life, promoting holistic development and creating a consistent environment that enhances their overall growth.”*

(p. 15).

Second, the administration also plays a significant role in providing educational alternatives. This involves ensuring that children in early intervention have access to quality education in the early stages. To achieve this, the inclusion of children in educational settings is promoted, and collaboration between early intervention professionals and educators is encouraged. This allows for the adaptation of educational environments and the development of specific intervention plans tailored to the individual needs of each child. Additionally, the administration can offer training programs and support for teachers to enhance their ability to cater to children with special needs, thus promoting a greater focus on family-centred planning.

*“Understanding family-centred planning has transformed my approach as an early intervention professional. Coordination with other contexts, such as social and community services, has enriched our interventions and opened up new opportunities to support families. By working collaboratively with other professionals, we have been able to provide families with additional resources and services that complement our work, thus strengthening the support network and improving outcomes for the child and their family.”*

(p. 20).

## 4. Discussion

The objective of this study was to describe and report on the experiences of professionals in early childhood intervention centres working with families of children with developmental disorders or at risk of having them who are served in these centres regarding how they implement FCP.

The first aspect discussed focuses on the personal conditions of parents seeking early intervention services for their children. Parents come with high expectations and distress due to a lack of information and the perception that time is crucial in treating their children. The news of a developmental disorder diagnosis is experienced as a mourning process for the loss of the idea of a perfect child. This leads parents to focus on rehabilitative treatment as the best option, as they want someone to do everything possible to help their children. However, this anxiety can lead to overprotection and the delegation of responsibilities to technicians, which is not healthy for both parents and children’s development [14]. Early intervention professionals feel responsible for attending to these families and recognize the need to address both the child and the family simultaneously. Parents’ expectations and their willingness to follow the professionals’ recommendations are high, which can increase the workload and stress for technicians [2].

The concern about session schedules and family flexibility is a prominent point in the passage. Although the literature acknowledges the importance of work–life balance, early childhood intervention services are generally expected to adapt to the needs and schedules of families [2,5]. The findings suggest a communication gap between early intervention professionals and parents. Communication could be improved through the implementation of effective education and guidance strategies for families to better understand the treatment process and set realistic expectations. It is crucial for these professionals to receive training and support in handling family expectations and emotions [19]. This could help alleviate concerns about blame and enhance the quality of care. To address scheduling and work–life balance concerns, a more family-centred approach could be considered, allowing greater flexibility in session schedules.

Participants also addressed the working conditions of technicians in early intervention centres. These professionals feel a significant responsibility, as their work involves commitment and dedication to assisting children with developmental disorders [2,13]. This can lead to stressful situations, especially when facing demands from parents and making treatment-related decisions. Professionals acknowledge the importance of teamwork and collaboration with other specialists, but at times, this can result in conflicts due to a lack of agreement on work objectives. Additionally, a lack of time to communicate with parents can create tensions and communication difficulties. Strategies to reduce workload, promote effective communication with parents, and foster a culture of teamwork and collaboration in these centres could be considered.

The third subtheme focuses on the shortcomings of the socio-health and educational systems, influencing the choice of a rehabilitative approach. Participants highlight the lack of uniformity in early intervention in Spain, with notable differences in how it is addressed across different autonomous communities (through social services or health). They also mention issues such as subsidies without quality review, a lack of resources and specialized professionals, waiting lists, and gaps between legislative and scientific recommendations. This reflects a common issue in many health and education systems, where regional disparities and a lack of resources can lead to inequities in the care of children with special needs [12,14].

Participants mention a paternalistic attitude and a lack of coordination between health and education professionals attending to the same child. This can lead to delays in intervention and negative consequences for the child’s development. The importance of coordination between health and education professionals in early intervention is a common theme in the literature [20,21]. The significance of collaboration among professionals from different fields to provide effective early intervention is emphasized. Studies have also suggested that a paternalistic attitude can be harmful, advocating for a rights-based approach for children [20].

Efforts are needed to unify early intervention approaches nationwide and establish quality standards [5]. This may require a review of legislation and increased investment in resources and professional training. Fostering collaboration between health and education professionals is also crucial. Coordination protocols can be established, and awareness can be promoted regarding the importance of early intervention. Professionals in health and education should receive training on the significance of early intervention and a rights-based approach. However, the lack of economic resources is a common issue in early intervention systems and can be a significant obstacle to implementing improvements [5].

However, there is also a socio-familial and work organization that promotes the implementation of FCP. Despite the mentioned barriers, the document emphasizes the importance of promoting family-centred planning, highlighting that both families and professionals, as well as the administration, can play a crucial role in its promotion [5]. Strategies to improve relationships and participation in the early intervention program are presented as essential. Parents can actively participate in the treatment program, offering valuable information about their children’s progress. They can also seek support to manage the situation and learn to cope with their children’s disorders. Collaboration between parents and professionals is fundamental [19].

Early intervention professionals play a vital role in promoting FCP. They must work closely with families, providing information, support, and necessary resources. Coordination with other professionals and services is also essential. The administration should play an active role in providing socio-health and educational alternatives, promoting coordination among different professionals and services, and supporting the inclusion of children in appropriate educational environments [5].

The findings presented align with the scientific literature in various aspects, such as the gap between actual and desirable practices [2]. Although professionals recognize the importance of family-centred practices, they may not always be able to fully implement them. This could be due to organizational barriers or a lack of resources. Studies also support the importance of collaboration between early intervention professionals and families in the development of children with disabilities or developmental disorders, as early assessment, counselling, and individualized planning are common practices in early intervention [7,8].

The findings suggest that, despite being aware of these responsibilities, professionals sometimes face difficulties in carrying out these practices effectively due to their workload. This may be a point of divergence from the literature, as specific work challenges can vary depending on the location and working context [22]. It would be interesting to consider the allocation of additional resources, such as more personnel or training in time management and teamwork. Additionally, professionals could benefit from professional development programs that help them acquire the skills and tools necessary to carry out family-centred practices effectively. Regular supervision and emotional support could help professionals deal with the frustrations and challenges they face in their work.

To improve the situation, a greater involvement of parents in the care and treatment of their children with disabilities could be encouraged, as highlighted by the participants, although this aspect is not found in the scientific literature. This could be achieved through educational programs that help parents better understand the needs of their children and provide them with tools to contribute more effectively to the treatment. This, coupled with support for family and work–life balance, can be addressed by promoting policies that facilitate the reconciliation of work and family care. This may include flexible work options and economic support measures for families.

In conclusion, the importance of FCP in early intervention for children with developmental disorders is emphasized. It is highlighted that, despite barriers and challenges, both families and professionals, as well as the administration, can play a crucial role in promoting a more family-centred approach. This involves a cultural shift in childcare, increased collaboration between parents and professionals, and more effective coordination among the different services and professionals involved in early intervention.

## Figures and Tables

**Table 1:** Interview protocol.

Interview Phase	Interview Topic	Content
Introduction	Motivation	Your experience offers a lesson that needs to be known by all.
	Information and ethical aspects	We need your consent to record the conversation. It will only be used for research purposes. We assure confidentiality, and only the researcher will have access to it. Your participation is voluntary, and you can interrupt or leave the interview at any time. After analysing the interviews, we will ask for your agreement with the transcriptions.
Beginning	Introductory general question	What is your experience in your workplace with families with children with developmental disorders or at risk of having them?
Development		The informants were encouraged to provide examples of their daily work related to family-centred planning.
Closure	Final question	Do you have anything to add?
	Appreciation	Thank you for the time you have dedicated to us, and please know that your testimony will be of great help.

**Table 2:** Example of an encoding process.

Citation	Initial Codes	Units of Meaning	Sub-Theme	Theme
“I have always had to do the reports in my own time. There is no time in the work schedule to do anything other than coordination (limited to two hours) and treatment with the children; there is no time to talk to the family” (p. 12).	The economy, no money for a master’s degree, working hours, working after hours, complaining, it is not my fault, more work, my boss calls me. Another child is on the schedule. I need an answer to the child’s problem now. I am not trained for this. It could be done differently.	Low pay, impossible hours, working out of hours, training on personal time, misdirected responsibility, parental complaints, stress, parental demands, lack of skills, lack of training, attitudinal barriers, wanting to do FCP but cannot.	Working conditions conducive to maintaining the rehabilitation model	A social and work organization that maintains rehabilitation or early stimulation practices.

**Table 3:** Sociodemographic data.

Participants	Gender	Age	Occupation	Time in Years Working in Early Childhood Care
P1	Man	26	Physiotherapy	3
P2	Woman	24	Speech therapy	3
P3	Woman	31	Psychology	7
P4	Man	25	Physiotherapy	2
P5	Woman	43	Speech therapy	1
P6	Woman	32	Psychology	5
P7	Woman	32	Psychology	5
P8	Woman	38	Speech therapy	6
P9	Man	31	Speech therapy	9
P10	Woman	36	Physiotherapy	10
P11	Woman	49	Psychology	11
P12	Woman	41	Psychology	5
P13	Woman	23	Speech therapy	1
P14	Woman	25	Physiotherapy	3
P15	Man	45	Psychology	10
P16	Man	32	Psychology	3
P17	Woman	36	Psychology	12
P18	Woman	27	Speech therapy	2
P19	Woman	29	Physiotherapy	5
P20	Woman	29	Speech therapy	5
P21	Woman	27	Psychology	1
P22	Woman	27	Psychology	4
P23	Woman	31	Speech therapy	3
P24	Woman	31	Physiotherapy	6
P25	Woman	26	Speech therapy	5

**Table 4:** Themes, sub-themes, and units of meaning derived from the research.

Themes	Sub-Themes	Units of Meaning
Social and work organizations that maintain rehabilitation or early stimulation practices.	Conditions of the person receiving early intervention services that lead to maintaining a rehabilitative model.	No reconciliation, depression, anxiety, guilt, physical deterioration, disbelief, frantic pace of life, need for sharing, burden on the mother, social stigma, session schedules, disrupting life patterns, or trauma.
	Working conditions conducive to maintaining the rehabilitation model	Underpaid, impossible schedules, working beyond regular hours, personal time used for training, misdirected responsibility, parental complaints, stress, parental demands, lack of skills, insufficient training, attitude barriers, and desire for family-centred planning but unable to implement.
	Shortcomings in the socio-healthcare and educational systems.	Dependency law, lack of economic resources, shortage of specialized professionals, waiting lists, impossible schedules, legislation without viable implementation, gaps between legislative and scientific recommendations, frustrated executives, and coordination problems.
Socio-family and work organizations that promote the implementation of family-centred planning	The role of the family in advocating for and preserving family-centred planning	Sharing childcare, cultural change, adaptation, sharing responsibilities, feeling useful, peace of mind, well-being, relying on professionals, seeking help, requesting to be heard, capable of providing information, and expressing a desire to participate in decision-making.
	The role of the professional in advocating for and preserving family-centred planning	Sharing information, family collaboration, coordination of contexts, parents participating in sessions, better if parents are in agreement, responsibility, feeling that my work is important, feeling capable, asking for help, seeking resources, or enjoying helping.
	The role of the administration: socio-sanitary alternatives to maximize family-centred planning	Legislation, salary improvements, service coordination, advocating for a model in management, improving work–life balance, financial assistance, home care, respite centres, and enhancement of medical care.

## Data Availability

The data presented in this study are available on request from the corresponding author. The data are not publicly available due to ethical reasons for confidentiality. The data are transcripts of interviews.
